# Molecular characterization of new described kobuvirus in dogs with diarrhea in China

**DOI:** 10.1186/s40064-016-3738-4

**Published:** 2016-11-30

**Authors:** Ning Kong, Yewen Zuo, Zhongze Wang, Hai Yu, En-min Zhou, Tongling Shan, Guangzhi Tong

**Affiliations:** 1 Department of Swine Infectious Disease, Shanghai Veterinary Research Institute, Chinese Academy of Agricultural Sciences, Shanghai, 200241 China; 2 Jiangsu Co-innovation Center for Prevention and Control of Important Animal Infectious Diseases and Zoonoses, Yangzhou, 225009 China; 3 Department of Preventive Veterinary Medicine, College of Veterinary Medicine, Northwest A&F University, Yangling, Shaanxi 712100 China

**Keywords:** Molecular characterization, Canine kobuvirus, Phylogenetic analysis

## Abstract

Canine kobuvirus (CaKVs) was a newly described virus detected in dogs in the US and Italy. To learn more about CaKVs, 5 of 106 fecal samples from diarrhea dogs were positive with CaKVs in China, and the full genome of CaKVs strain CH-1 isolated from dog with diarrhea was sequenced. The genome consists of 8186 nucleotides, excluding the 3′ poly (A) tail, and an open reading frame that maps between nucleotide positions 601 and 7943 which encodes a 2446 amino acid polyprotein. Based on the complete amino acid sequence of polyprotein, phylogenetic analysis showed that CH-1 was grouped along with other canine kobuvirus strains detected in the USA (US-PC0082, AN211D).

## Background

Picornaviruses belonging to Picornaviridae family are small, nonenveloped viruses with a single-stranded positive-sense RNA genome encoding a single polyprotein, which is genetically highly diverse and infects a wide range of vertebrate species including human and animal. The Kobuvirus which was identified as a newly genus in the family Picornaviridae, consists of three species, Aichivirus A (formerly Aichi virus) (Yamashita et al. 1998), Aichivirus B (formerly Bovine kobuvirus) (Yamashita et al. 2003) and Aichivirus C (porcine kobuvirus) (Reuter et al. 2009). The species Aichivirus A consists of four types: Aichi virus 1, canine kobuvirus 1 (Kapoor et al. 2011; Li et al. 2011), feline kobuvirus 1 (Chung et al. 2013) and murine kobuvirus 1 (Phan et al. 2011). The species Aichivirus B consists of three types: bovine kobuvirus 1 (Yamashita et al. 2003), ferret kobuvirus 1 (Smits et al. 2013) and ovine kobuvirus (Reuter et al. 2010). The species Aichivirus C consists of a single type: porcine kobuvirus 1 (Reuter et al. 2008). Recently, a distinct group of kobuviruses, designated caprine kobuviruses (CKVs) was proposed as a new candidate species, Aichivirus D, within the genus Kobuvirus (Melegari et al. 2016; Oem et al. 2014). The host range of CaKV included four species in the canid species (domestic dog, red fox, golden jackal and side-striped jackal) and one non-canid species (spotted hyena, family Hyaenidae) (Di Martino et al. 2014; Olarte-Castillo et al. 2015). As companion animals, dog contacting with human frequently may be a potential intermediary to translate zoonotic virus. More recently, Canine kobuvirus have been recently discovered in dogs in USA, UK and Italy (Carmona-Vicente et al. 2013; Di Martino et al. 2013; Kapoor et al. 2011; Li et al. 2011). To extend these initial findings, we detected this newly characterized virus in stool samples from dogs with diarrhea in China, and sequenced the nearly complete genome of one isolate virus, CH-1.

## Results and discussion

Out of 106 faecal samples, 5 (4.7%) were positive for the newly described CaKVs detected using reverse transcription-PCR (RT-PCR). Sequence analysis of the partial region, based on the 598-bp sequences, showed that the 5 sequences shared 98.3–99.8% identity with each other, suggesting that they could be considered members of the same virus species. The five sequences share 93–94% sequence identities with the CaKVs (CaKV/PC0082/US, CaKV/AN211D/US and CaKV/UK003/US) isolated from USA, respectively.

**Fig. 1:**
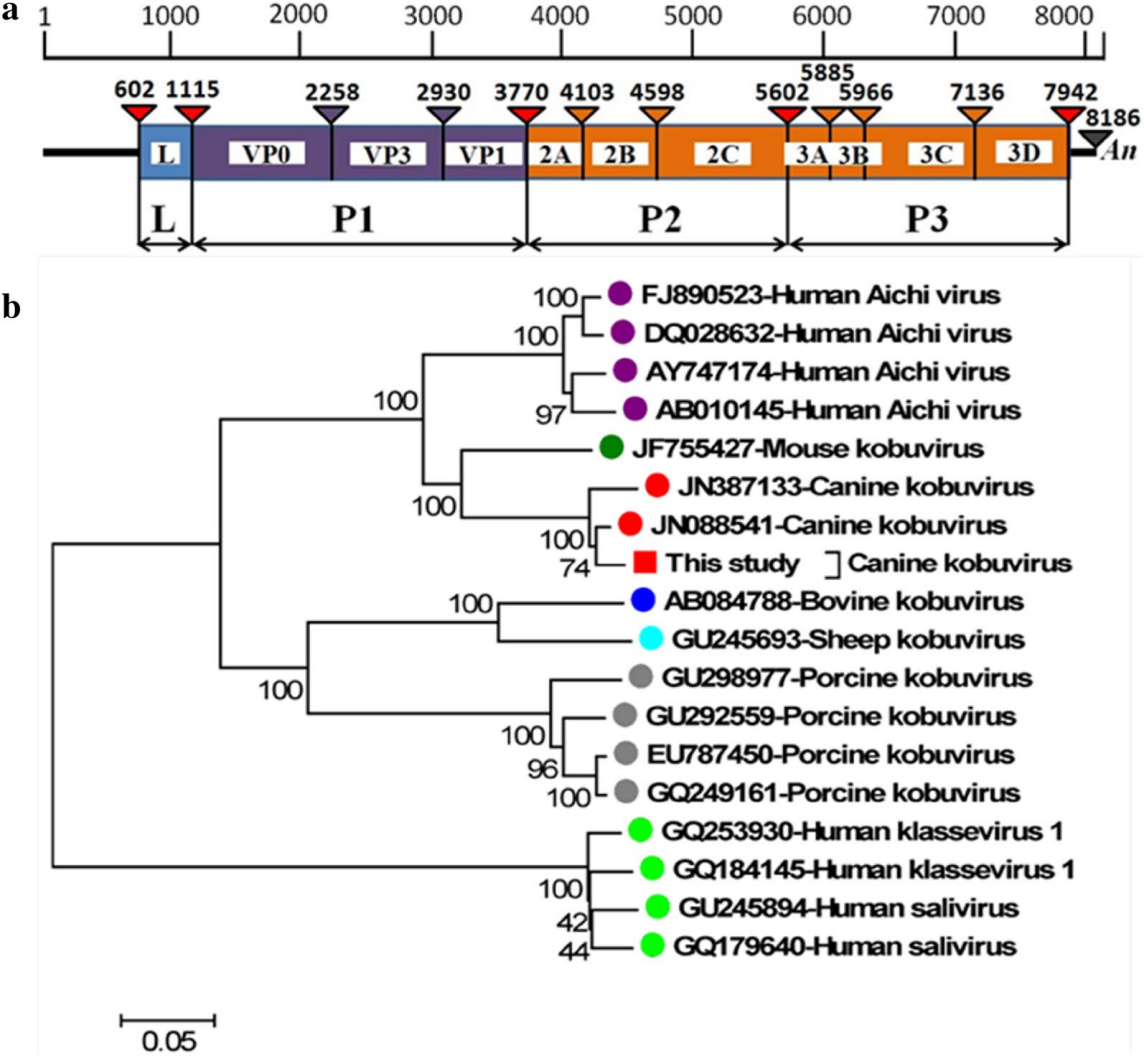
**a** Genome organization of CaKV. **b** Phylogenetic tree was constructed by the neighbour-joining method with p-distances and 1000 bootstrap replicates using MEGA4.0 software (www. megasoftware.net) with an alignment of the complete amino acid sequence of polyprotein with known kobuviruses and kobu-like viruses. GenBank accession numbers are indicated. The isolate of CH-1 is marked with a *square*. *Scale bar* indicates estimated phylogenetic divergence

The complete genome of one strain CaKVs named CH-1, was detected and sequenced using 6 pair of primers designed basing on the genome of CaKV/AN211D/US (GenBank ID no. JN387133), which was 8186 nt and containing a partial 5′UTR of 601 nt, an open reading frame (ORF) with a length of 7341 nt encoding a putative polyprotein precursor of 2446 aa, and a 3′UTR of 244 nt (not containing poly A). The near-full genome of CH-1 shares a high G+C content (58.6%) with kobuviruses (52–59%).The putative polyprotein precursor encodes leader protein L (171 aa), capsid proteins VP0 (381 aa), VP3 (224 aa), VP1 (280 aa) and nonstructural proteins 2A (111 aa), 2B (165 aa), 2C (335 aa), 3A (94 aa), 3B (27 aa), 3C (390 aa) and 3D (269 aa) (Fig. 1a). The viral genome sequence described here has been deposited in the GenBank database and assigned accession no. JQ911763.

CH-1 showed 94% nucleotide identity with CaKVs genomes of US-PC0082 (JN088541), AN211D (JN387133) and UK003 (KC161964), respectively. Phylogenetic analysis using the complete polyprotein of CH-1 and 17 representative kobuvirus and related viruses was performed (Fig. 1b). In the tree, the strain CH-1 was grouped along with other canine kobuvirus strains identified in the USA (US-PC0082, AN211D). This finding showed that the newly described kobuvirus, CaKVs, might be widespread distribution in China.

## Methods

This study was carried out in strict accordance with the recommendations in the guide for the care and use of laboratory animals by the authority of the People’s Republic of China. With approval from the animal ethics committee at Shanghai Veterinary Research Institute, Chinese Academy of Agricultural Sciences for the protocol (Permit Number: shvri-do-0330). During October 2010–April 2011, fecal samples were collected from 106 dogs with diarrhea in People’s Republic of China. Stool samples were suspended to 10% (wt/vol) in phosphate-buffered saline (0.01 M, pH7.4), and vigorously vortexed for 5 min. Two hundred microliters of supernatant was collected after centrifugation (5 min, 12,000*g*) for Extracting Viral nucleic acids using the QIAamp viral RNA extraction kit (Qiagen). Viral RNA was dissolved in 50 μL RNase-free water and stored at −80 °C.

Based on the kobuvirus genomes (GenBank No.JN387133), primers were designed for detecting the virus and cloning the full genomes of the viruses. Primers (CaKVs-L1 5′-ATACTTCACTGCCCAAACCC-3′, CaKVs-R1 5′-GAAGGTGAGTGGGGACAAGG-3′, CaKVs-L2 5′-AGACCGCGATTCCATCTGGA-3′ and CaKVs-R2 5′-GAGGTAGCGGGAGAAGATGT-3′) were used for reverse transcription nested PCR to detected CaKVs by amplification of a 598-bp fragment. The expected size DNA bands were excised from an agarose gel, purified with the AxyPrep DNA gel extraction kit (Axygen, Union City, CA, USA), cloned into pMD-18T vector (TaKaRa, Dalian, China), and sequenced (Applied Biosystems 3730 DNA Analyzer; Invitrogen).

The complete genome of CH-1, was detected and sequenced using 6 pair primers (Table 1) designed based on the Canine kobuvirus mRNA sequence (GenBank ID nos. JN387133) deposited in the GenBank database (NCBI) by polymerase chain reaction (PCR). The 5′ and 3′ ends of the CaKVs genome were amplified by the SMART RACE cDNA amplification kit (Clontech, China). PCR was performed using pfu DNA polymerase (Thermo Fisher Scientific). Each of the PCR products was sequenced and assembled using SeqMan software to obtain the full genome of CaKVs. The open reading frame (ORF) was identificated by Open Reading Frame Finder (ORF Finder) (http://www.ncbi.nlm.nih.gov/gorf/gorf.html). Multiple sequence alignment were performed using CLUSTALW software and phylogenetic trees were generated by the neighbor joining (NJ) method with bootstrap of 1000 replicates using MEGA 4.0 software.

**Table 1:** List of primers used in this study

Primer names	Sequence of primers (5′–3′)	Position (nt)
5′ race reverse-1	CAGTAGCAGTGATGTAGGTGAGGC	640
5′ race reverse-2	GCGGTAGAAAGAGTTCACAGTGG	618
5′ race reverse-3	CGCACAGAACGAGATTCCATT	593
3′ race forward-1	CGGGTTCGCTCCAAATTTC	7821
3′ race forward-2	TACCCTACTCCTACCTCCAACTCC	7841
3′ race forward-3	CGCTGGCTCAACCTGCTG	7864
1 forward	CCACTCTAATACCCCGAGGAAT	448
1 reverse	AGGTACGGTCACCGGAAGGT	1618
2 forward	CGAAGGAGCCGGGAAACT	1371
2 reverse	GGCAATCAGGACAGAGGTGG	3256
3 forward	ACTCTGCCCCCGCCTCTGCCCTCAT	2798
3 reverse	AGCCAAGTAAGGAAGGACACAAC	4328
4 forward	ACTGTACCCACTTTGTCCAAGGT	3911
4 reverse	AGTTCATCAGCGTAAGCTTCAGG	5609
5 forward	CCATGAACCCAACGAACGC	5238
5 reverse	CGATTTCGACTTGGAGTTCGG	6916
6 forward	CCATCAAGAAGGAACCGGC	6575
6 reverse	GTGGGTGGTCTTTATATCACCATG	7982

## Conclusions

The present study showed that CaKVs were detected in fecal samples of dogs with diarrhea in China. Analysis the complete genome of one strain CaKVs named CH-1 found that CH-1 was grouped along with other canine kobuvirus strains detected in the USA (US-PC0082, AN211D). The epidemiologic surveillance and genome characterization of CaKVs might help clarify the global distribution and possible association of CaKVs with enteric diseases in dogs.
